# Chemical Analysis and *In Vitro* Bioactivity of Essential Oil of *Laurelia sempervirens* and Safrole Derivatives against Oomycete Fish Pathogens

**DOI:** 10.3390/molecules26216551

**Published:** 2021-10-29

**Authors:** Alejandro Madrid, Ana Lizeth Morales, Valentina Saffirio, Mauricio A. Cuellar, Enrique Werner, Bastián Said, Patricio Godoy, Nelson Caro, Mirna Melo, Iván Montenegro

**Affiliations:** 1 Laboratorio de Productos Naturales y Síntesis Orgánica (LPNSO), Departamento de Ciencias y Geografía, Facultad de Ciencias Naturales y Exactas, Universidad de Playa Ancha, Avda. Leopoldo Carvallo 270, Playa Ancha, Valparaíso 2340000, Chile; ana.abularach1995@gmail.com (A.L.M.); valentina.saffirio@gmail.com (V.S.); 2 Facultad de Farmacia, Escuela de Química y Farmacia, Universidad de Valparaíso, Av. Gran Bretaña 1093, Valparaíso 2340000, Chile; mauricio.cuellar@uv.cl; 3 Departamento de Ciencias Básicas, Campus Fernando May, Universidad del Bío-Bío, Avda. Andrés Bello 720, Casilla 447, Chillán 3780000, Chile; ewerner@ubiobio.cl; 4 Departamento de Química, Universidad Técnica Federico Santa María, Av. Santa María 6400, Vitacura, Santiago 7630000, Chile; bastian.said@usm.cl; 5 Facultad de Medicina, Instituto de Microbiología Clínica, Universidad Austral de Chile, Los Laureles s/n, Isla Teja, Valdivia 5090000, Chile; patricio.godoy@uach.cl; 6 Centro de Investigación Austral Biotech, Facultad de Ciencias, Universidad Santo Tomás, Avda. Ejército 146, Santiago 8320000, Chile; ncaro@australbiotech.cl; 7 Instituto de Química, Facultad de Ciencias, Pontificia Universidad Católica de Valparaíso, Av. Universidad #330, Curauma, Valparaíso 2340000, Chile; mirna.melo.f@mail.pucv.cl; 8 Facultad de Medicina, Escuela de Obstetricia y Puericultura, Universidad de Valparaíso, Angamos 655, Reñaca, Viña del Mar 2520000, Chile

**Keywords:** *Laurelia sempervirens*, safrol, saprolegniosis, nitrocompounds

## Abstract

In this study, the essential oil (EO) from *Laurelia sempervirens* was analyzed by GC/MS and safrole (**1**) was identified as the major metabolite **1**, was subjected to direct reactions on the oxygenated groups in the aromatic ring and in the side chain, and eight compounds (**4** to **12**) were obtained by the process. EO and compounds **4**–**12** were subjected to biological assays on 24 strains of the genus *Saprolegnia*, specifically of the species 12 *S.* *parasitica* and 12 *S.* *australis*. EO showed a significant effect against *Saprolegnia* strains. Compound **6** presents the highest activity against two resistant strains, with minimum inhibitory concentration (MIC) and minimum oomyceticidal concentration (MOC) values of 25 to 100 and 75 to 125 µg/mL, respectively. The results show that compound **6** exhibited superior activities compared to the commercial controls bronopol and azoxystrobin used to combat these pathogens.

## 1. Introduction

Over the last decades, the production of fish, crustaceans, shellfish, and amphibians through aquaculture has become the fastest growing food sector in the world. However, the growing business of aquaculture often suffers from heavy financial losses due to the development of infections caused by microbial pathogens, particularly by different species of the genus *Saprolegnia* [1]. The infection caused by this genus of pathogens is known as saprolegniosis; this infection occurs in all freshwater aquatic ecosystems and significantly attacks fish and eggs, mainly when certain changes occur in the culture environment, which causes the appearance of these oomycetes [2]. The mechanism of infection begins by means of its spores, which infect the epidermal tissues of fish, generally starting from the head and fins, spreading over the entire surface of the body, causing cell necrosis with cutaneous and epidermal damage. The oomycete penetrates the membrane affecting the ova, and proliferation occurs through the water to other closer ones, causing death [3]. Until 2002, *Saprolegnia* sp. was kept under control through the use of Malachite green; however, due to its carcinogenic and toxicological effects, treatment with this chemical has been banned internationally [4]. Formalin is another of the most potent fish oomycide, but with the drawback of having an acute impact on aquatic ecosystems [5]. Other chemical alternatives have been used to control the disease effectively, including 8-quinolinol, dichlorophen, copper sulfate pentahydrate, sodium chloride or bronopol [6,7,8]. However, to date, most of the compounds described for the control of *Saprolegnia* are ineffective or have negative effects on the health of fish, operators or the environment [9]. Hence, an economical, effective and friendly alternative is required.

Not insignificant is the recent increased interest in using natural substances derived from plants for different industrial purposes, not only as food seasoning or natural medicine, but also for pest management in agriculture, livestock, and aquaculture. The plant-derived products most commonly used to control diseases are the essential oils. These are complex mixtures of hydrocarbons and oxygenated hydrocarbons arising from the isoprenoid pathways, including terpenoids and phenylpropanoids [10]. These compounds have served as the starting point for the discovery and development of new antimicrobial agents.

The genus *Laurelia* belongs to the family Atherospermataceae, is represented by only two species, and is endemic to the southern hemisphere. Both species have a high content of alkaloids, and other compounds include terpenes, phenolics, phenylpropanoids, and flavonoids, which usually have a wide range of pharmacological activities [11,12].

*Laurelia sempervirens* (Ruiz and Pav.) Tul. is an evergreen endemic tree of the temperate rainforests of southern Chile [13]. Numerous pharmacological and phytochemical studies have reported repellent, insecticidal, antibacterial, antioxidant, antitumoral and antifungal activity potential of essential oil from *L. sempervirens* [14,15,16,17]. 

Safrole is a major component of Chilean laurel oil and a component of several other essential oils [18,19]. It has differential biological activities, such as cytotoxic, analgesic and antimicrobial activities [20], which can be used to participate in numerous chemical reactions.

Therefore, our working group has developed safrole derivatives, by direct reactions on oxygenated groups in the aromatic ring and the side chain, in search of new biological activities such as antioomycete.

The objective of this study was to evaluate the antioomycete activity of the essential oil of *L. sempervirens*, its main compound safrole, and its synthetic derivatives against strains of *S. parasitica* and *S. australis*, both pathogenic oomycetes of economic importance for salmon farming.

## 2. Results and Discussion

### 2.1. Yield and Chemical Constituents of EO

The extraction yield of *L. sempervirens* EO was 2.78% (v/dry weight) and the density was 1.15 ± 0.01 g/mL. The results of the gas chromatographic analysis of *L. sempervirens* EO is summarized in Table 1.

**Figure 1:**
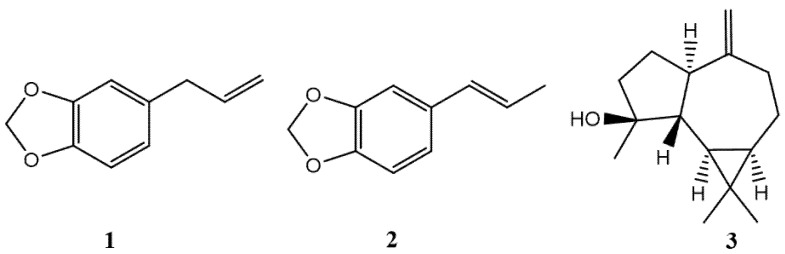
Structures of the main compounds present in the EO of *L. sempervirens*.

Sixteen components were identified in the leaf EO: 78.06% were phenylpropanoids, 3.08% terpenes and 0.37% benzaldehydes. Leaf EO was mainly characterized by safrole (**1**) (65.03%), isosafrole (**2**) (11.90%), and spathulenol (**3**) (11.16%) (see Figure 1). 

Some studies of leaf EOs from *L. sempervirens* showed different chemical compositions according to the region. The results of this work show that the chemical composition of this EO was different with those obtained from plants collected in the Metropolitan, Ñuble, Bio-Bío and La Araucanía regions [14,16,17,22]. The most important differences are found in the absence of spatulenol content, the lower isosafrole content, and a higher safrole content. In this context, the variability in the essential oil content is influenced by the geographical origin of the species, as well as by the drying methods, the extraction time and the type of organ studied [23].

Safrole derivatives can be synthesized by chemical modification, and some of these derivatives have been proved to have better antimicrobial activity; indeed, in the last decade, this research group reported some safrole derivatives obtained by traditional organic chemistry protocols with a series of modifications [24,25]. 

In this work, however, the preparation of catechol from the methylenedioxy ring safrole cleavage reaction was performed with TiCl_4_ to shorten the reaction time and improve the yield, in comparison with the previously reported method [25].

**Figure 2:**
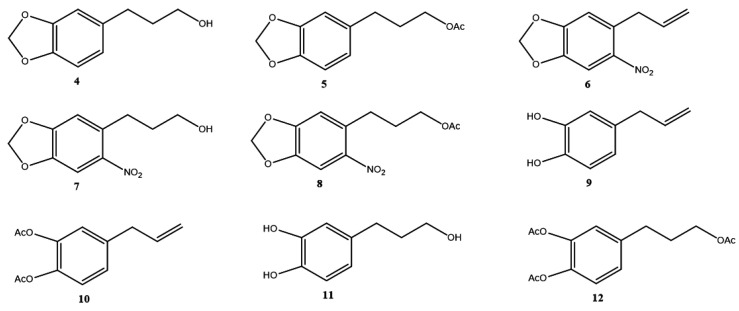
Structure of safrole derivatives **4**–**12**.

Safrole derivatives **4**–**12** were obtained in moderate to good yields (40.0–95.0%) (Figure 2). NMR data of **4**–**12** were consistent with what we had previously reported (see Supplementary Materials).

### 2.2. Biological Assays

The in vitro antimycotic activity of *L. sempervirens* EO, safrole and its derivatives **4**–**12** against twenty-four strains of *S. parasitica* and *S. australis*, twelve of each.

First, the minimum inhibitory concentration (MIC) at 48 h of EO, safrole and its derivatives **4**–**7** (Table 2) and for compounds **8**–**12** (Table 3) was evaluated against 22 strains of *Saprolegnia* (*S. parasitica* 1–11 and *S. australis* 12–22, respectively).

EOs used in this study presented activity against the 22 isolates tested. The highest MIC value observed for EO was against strain 1 of *S. parasitica* and strain 18 of *S. australis*, with 50 µg/mL, while the minimum value was of 175 µg/mL for strain 15 of *S. australis*. When comparing the effectiveness of the oil against the strains, it was observed that EOs were very efficient against the pathogenic strain of *S. parasitica* on the strains of *S. australis*. EO showed bronopol-equivalent effectiveness against strains 1, 11, 17 and 20, while azoxystrobin showed an equivalence in the action on the strains 6, 7 and 11. 

However, the EO showed higher anti-oomycete activity against strains 21 and 22 and 1, 2, 3, 4, and 8, for bronopol and azoxystrobin, respectively, but both controls showed lower efficiency than EO against strains 18 and 19 of *S. australis*. As an aside, previous studies have reported that EOs from *Laureliopsis philippianna*, *Tymus vulgaris* and *Origanum vulgare* present similar anti-oomycidal activity against *S. parasitica*, due to their high content of aromatic compounds [23,26].

For safrole and its derivatives, the results show that **6** and **7** were the most active compounds compared to the controls against the twenty-two strains tested. However, the MIC values of compound **6** for the growth inhibition of *S. parasitica* and *S. australis* were 3.125 to 6.25 and 6.25 to 75 µg/mL, respectively, while for compound **7**, which differs in the presence of a primary alcohol positioned on the side chain of the aromatic ring with **6**, these values decreased from 3.125 to 6.25 and 50 to 125 µg/mL for the strains, respectively. These data confirm what has been suggested in previous studies about the importance that exists between nitro groups and primary alcohols in the sense that they can generate intra-molecular hydrogen bridges that would diminish the antimicrobial action of this type of molecule [27].

Subsequently, the oil and all the compounds were evaluated against strains 23 and 24, corresponding to *S. parasitica* and *S. australis*, respectively, which were less sensitive to the commercial products tested, such us fluconazole and ketoconazole.

Table 4 summarizes the MIC, the minimum oomyceticidal concentration (MOC) and membrane damage values for the EO, safrole and its derivatives **4**–**12** against strains 23 and 24.

Our results show that nitrosylated compounds can cause greater damage to *Saprolegnia* strains when compared to commercial antifungal compounds (bronopol and azoxystrobin) used as controls.

In a biological system, nitro groups can be enzymatically reduced, causing very unstable nitro radicals that can be re-oxidized under aerobic conditions, generating reactive superoxide anions. However, a reduction in the NO_2_ groups can also originate nitroso and hydroxylamine intermediates that react with biomolecules to produce toxic effects [28], including protein cysteine thiols [29]. On the other hand, we can consider binding interactions between nitroaromatics compounds and biomacromolecules such as proteins, because nitrobenzene compounds can act as enzymatic inhibitor by interaction with tryptophan and tyrosine residues [30]. This supports the importance of specific interactions of nitro aromatic compounds to cause cell membrane proteins’ malfunction and cell membrane damage.

Nitrosylation increased the efficiency of safrole derivatives, improving their antifungal activity reflected in the damage to cell membranes. These good results for safrole-derived compounds open the possibility that they could be considered as effective control agents, including animal and plant pathogenic oomycetes, because previous studies demonstrated their low toxicity in *in vitro* models of healthy epithelial cells, red blood cells and *Artemia salina* [25,31].

## 3. Materials and Methods

### 3.1. General

Standard pure compounds for co-injection in GC/MS, α-pinene, β-phellandrene, safrole, isosafrole, and piperonal were purchased from Sigma-Aldrich Co. (St. Louis, MO, USA). The separation and identification of the synthesized compounds was carried out by previously performed methods [24,25].

### 3.2. Plant Material

The leaves of *L. sempervirens* were collected in Antilhue, Los Ríos Region, Chile, in December 2019. The plant material was identified by Forestry Engineer Patricio Novoa (National Botanical Garden, Viña del Mar, Chile), and a voucher specimen (LS-1219) was deposited at the Natural Products and Organic Synthesis Laboratory of Universidad de Playa Ancha, Valparaíso, Chile.

### 3.3. Essential Oil Isolation and Analysis

The fresh leaves (500 g) of *L. sempervirens* were submitted to hydrodistillation for 4 h. The distilled leaves were extracted using ethyl acetate, dried over anhydrous sodium sulfate, and the solvent evaporated. The EO was analyzed and its components identified by gas chromatography/mass spectrometry (GC/MS) using previously standardized instrumentation and methodology [32].

### 3.4. Isolation and Identification of the Major Constituent

EO obtained as described above (5.0 g) was chromatographed on a column of silica gel and eluted with a gradient of hexane/ethyl acetate (100:0 to 90:10). One hundred and fifty fractions were collected and analyzed by TLC. The fractions 18–78 were purified by flash chromatography using a mixture of hexane/ethyl acetate (95:5) as eluent. Thirty fractions were collected. Fractions 13–25 contained the active compound (2.1 g) identified as safrole (**1**).

### 3.5. Synthesis of Safrole Derivatives

Safrole derivatives (Figure 2) obtained from the natural compound **1** were synthesized and characterized by standard methods [24,25], except for compound **9**, 4-allylbenzene-1,2-diol.

The natural compound **1** (100 mg, 0.62 mmol) was added to a stirred solution of TiCl_4_ (0.3 mL, 0.098 mmol) in dry dichloromethane (10 mL) under nitrogen atmosphere. The mixture was stirred for 8 h at room temperature, diluted with water (20 mL), extracted with ethyl acetate (2 × 50 mL), dried (Na_2_SO_4_), and concentrated. The separation and identification of the compound **9** was carried out by the previously performed method [25].

### 3.6. Oomycete Strain

Twelve *Saprolegnia parasitica* strains and twelve *S. australis* strains (the Cell Biology Laboratory, Faculty of medicine, Universidad de Valparaíso) were used in this study. These were isolated from *Salmo salar* carp eggs and biochemically and molecularly characterized in previous studies [23].

### 3.7. Minimum Inhibitory Concentration Evaluation

Minimum inhibitory concentration (MIC) was determined by a serial dilution technique using 96-well microtiter plates. The EO, safrole (**1**) and compounds **4**–**12** were dissolved in 0.1% DMSO solution and added to a Gypsum (G-Y) medium with inoculum. Samples were evaluated at a series of previously defined concentrations [23]. The microplates were incubated in a rotary agitator (160 rpm) for 48 h at 20 °C. The lowest concentrations without visible mycelia growth under an optical microscope were defined as the concentrations that completely inhibited oomycete growth according to the above standardized method [23].

### 3.8. Spores Germination Inhibition Assay

The minimum oomyceticidal concentration (MOC) was determined by the agar dilution method [33]. Briefly, 10 μL serial sub-cultivation of the tested compound was dissolved in a cultivation medium and inoculated during 72 h in microtiter plates containing 100 μL of broth per well and with incubation for 72 h at 25 °C. The lowest concentration without visible growth or germination of spores was defined as MOC, indicating the death of 99.5% of the original. The commercial oomycides bronopol and azoxystrobin were used as positive controls.

### 3.9. Cell Membrane Damage Measurement (Cellular Leakage Assay)

Cell leakages were measured in order to determine the effectiveness of the EO, safrole and compounds **4**–**12** on membrane integrity. This method was assessed according to Flores [34].

### 3.10. Statistical Analysis

The statistical data of recovery rates were performed by comparison within isolates and between culturing media following a standard method [34].

## 4. Conclusions

In summary, sixteen compounds were identified from the EO of *L. sempervirens*. Safrole (**1**) is the major metabolite detected by GC/mass spectrometry. Compounds **4**–**12** were biologically active against *Saprolegnia*. Significant antioomycete activities of novel compounds **5**, **6**, **7** and **8** were observed against *S. parasitica* and *S. australis*. This study provides baseline information on the potential application of EOs as botanical fungicides in fish farms.

## Figures and Tables

**Table 1:** EO composition of leaves of *L. sempervirens*.

N	RT (min)	Components	% Area ^a^	RI ^b^	RI ^c^	Identification
1	10.22	α-Pinene	1.48	941	941	RL, MS, Co
2	10.24	Camphene	0.11	956	957	RL, MS
3	10.32	α-Phellandrene	0.33	1010	1009	RL, MS
4	10.60	p-Cymene	0.12	1032	1033	RL, MS
5	10.77	β-Phellandrene	1.08	1038	1035	RL, MS, Co
6	10.83	3-Carene	0.42	1147	1148	RL, MS
7	10.88	Isosafrole	11.90	1227	1229	RL, MS, Co
8	11.04	Safrole	65.03	1293	1295	RL, MS, Co
9	11.13	Piperonal	0.37	1348	1347	RL, MS, Co
10	11.94	3-Allyl-6-methoxyphenol	1.13	1360	1362	RL, MS
11	12.26	β-Caryophyllene	0.74	1395	1396	RL, MS
12	12.52	α-Gurjunene	0.24	1397	1399	RL, MS
13	15.83	Humelene	0.35	1441	1442	RL, MS
14	15.90	Germacrene D	2.33	1480	1480	RL, MS
15	16.05	Spathulenol	11.16	1560	1562	RL, MS
16	16.10	α-Cadinol	1.10	1615	1615	RL, MS
		Total identified	97.89			

^a^ Surface area of GC peak; ^b^ RI: retention indices relative to C_8_-C_36_ *n*-alkanes on the HP-5 MS capillary column; ^c^ RI: retention index from the literature [20]. RL: comparison of the RI with those of the literature [21]; MS: comparison of the mass spectra with those of the NIST 14; Co: co-elution with standard compounds available in our laboratory.

**Table 2:** MIC values ^a^ of leaf EO, safrole and compounds **4**–**7** against mycelium at 48 h.

N Strains			MIC (µg/mL)						
	EO	1	4	5	6	7	Bronopol	Azoxystrobin	DMSO
1	50	125	175	3.125	3.125	3.125	50	100	i
2	75	100	150	6.25	6.25	6.25	50	100	i
3	75	125	150	6.25	6.25	6.25	50	100	i
4	75	100	150	12.5	3.125	12.5	50	100	i
5	100	125	175	25	6.25	25	50	75	i
6	125	175	175	50	6.25	50	75	125	i
7	100	150	175	50	3.125	50	50	100	i
8	100	150	200	50	6.25	50	75	150	i
9	125	150	150	50	6.25	50	50	100	i
10	125	150	>200	50	3.125	50	100	100	i
11	100	150	>200	50	6.25	50	100	100	i
12	125	175	>200	50	6.25	50	100	100	i
13	150	175	>200	50	12.5	50	100	100	i
14	150	175	200	50	25	50	100	100	i
15	175	200	>200	25	25	50	100	100	i
16	125	150	>200	25	50	75	50	100	i
17	75	125	175	12.5	25	25	75	100	i
18	50	100	150	6.25	50	75	100	100	i
19	75	125	150	50	12.5	50	125	100	i
20	150	175	>200	100	50	100	150	125	i
21	125	150	>200	100	50	100	150	100	i
22	125	175	>200	125	75	125	150	100	i

^a^ Each value represents the mean of three experiments, performed in quadruplicate. i: inactive.

**Table 3:** MIC values ^a^ of compounds **8**–**12** against mycelium at 48 h.

N Strains	MIC (µg/mL)							
	8	9	10	11	12	Bronopol	Azoxystrobin	DMSO
1	12.5	50	125	150	100	50	100	i
2	12.5	50	125	150	100	50	100	i
3	125	75	100	150	100	50	100	i
4	150	75	125	175	100	50	100	i
5	150	100	150	150	125	50	75	i
6	175	75	125	175	125	75	125	i
7	150	50	150	200	150	50	100	i
8	>200	100	200	175	150	75	150	i
9	125	50	125	175	150	50	100	i
10	175	75	200	150	175	100	100	i
11	175	175	200	>200	200	100	100	i
12	125	150	150	200	>200	100	100	i
13	150	150	175	>200	150	100	100	i
14	175	175	175	>200	125	100	100	i
15	175	75	200	>200	200	100	100	i
16	>200	50	>200	200	>200	50	100	i
17	125	50	175	150	175	75	100	i
18	175	50	175	>200	200	100	100	i
19	100	50	125	150	150	125	100	i
20	200	50	200	175	175	150	125	i
21	175	175	175	150	125	150	100	i
22	175	175	175	150	125	150	100	i

^a^ Each value represents the mean of three experiments, performed in quadruplicate. i: inactive.

**Table 4:** Summary of the anti-oomycete activity of EO, safrole and derivatives against resistant strains of *S*. *parasitica* and *S. australis*.

Compound	*S. parasítica* (Strain 23)			*S. australis* (Strain 24)		
	MIC (µg*·*mL^−1^) ^a^	MOC (µg*·*mL^−1^) ^a^	Membrane Damage (%) ^b^	MIC (µg*·*mL^−1^) ^a^	MOC (µg*·*mL^−1^) ^a^	Membrane Damage (%) ^b^
EO	50	75	86.3 ± 0.4	75	75	84.3 ± 0.9
1	125	150	75.4 ± 0.6	150	150	72.4 ± 1.1
4	150	175	6.3 ± 0.4	175	175	5.9 ± 0.4
5	150	175	80.3 ± 0.2	175	200	50.5 ± 0.6
6	25	75	96.3 ± 0.3	100	125	90.6 ± 0.9
7	100	125	77.4 ± 0.7	150	175	60.8 ± 0.4
8	125	150	33.4 ± 0.6	175	200	27.5 ± 0.7
9	100	150	55.8 ± 0.4	150	175	40.4 ± 0.8
10	150	175	17.4 ± 0.1	175	200	12.2 ± 0.4
11	200	>200	0	>200	>200	0
12	150	175	25.6 ± 0.4	>200	>200	21.4 ± 0.3
Bronopol	50	100	27.1 ± 0.3	100	150	26.0 ± 0.4
Azoxystrobin	100	125	Nd	100	150	Nd
SDS	-	-	100	-	-	100
DMSO	i	i	i	i	i	i

^a^ Each value represents the mean of three experiments. ^b^ Each value represents the mean ± SD of three experiments, performed in quadruplicate. Nd: not detected; i: inactive.

## Data Availability

All data are available for the scientific community.

## Supplementary Materials

The following are available online. NMR data of known compounds **1**, **4**–**12**.

## Sample Availability

Samples of the essential oil and compounds are available from the authors.
