# Supplementary material for: Chemical Analysis and In Vitro Bioactivity of Essential Oil of Laurelia sempervirens and Safrole Derivatives against Oomycete Fish Pathogens
Source: Molecules. 2021 Oct 29;26(21):6551. doi: 10.3390/molecules26216551 (PMC8588113; doi:10.3390/molecules26216551)
Supplement: Supplementary file 1 [file molecules-26-06551-s001.zip › molecules-1434778-supplementary.pdf]

SUPPORTING INFORMATION

Chemical Analysis and *In Vitro* Bioactivity of Essential Oil of *Laurelia sempervirens* and Safrole Derivatives against Oomycete Fish Pathogens

Alejandro Madrid [1, *], Ana Lizeth Morales [1]; Valentina Saffirio [1], Mauricio A. Cuellar [2], Enrique Werner [3], Bastián Said [4], Patricio Godoy [5], Nelson Caro [6], Mirna Melo [7] and Iván Montenegro [8*]

1 Laboratorio de Productos Naturales y Síntesis Orgánica (LPNSO), Departamento de Ciencias y Geografía, Facultad de Ciencias Naturales y Exactas, Universidad de Playa Ancha, Avda. Leopoldo Carvallo 270, Playa Ancha, Valparaíso 2340000, Chile; alejandro.madrid@upla.cl

2 Facultad de Farmacia, Escuela de Química y Farmacia, Universidad de Valparaíso, Av. Gran Bretaña 1093, Valparaíso 2340000, Chile; mauricio.cuellar@uv.cl

3 Departamento de Ciencias Básicas, Campus Fernando May, Universidad del Bío-Bío. Avda. Andrés Bello 720, casilla 447, Chillán 3780000, Chile; ewerner@ubiobio.cl

4 Departamento de Química, Universidad Técnica Federico Santa María, Av. Santa María 6400, Vitacura 7630000, Santiago, Chile; bastian.said@usm.cl

5 Instituto de Microbiología Clínica, Facultad de Medicina, Universidad Austral de Chile, Los Laureles s/n, Isla Teja, Valdivia 5090000, Chile; E-Mails: patricio.godoy@uach.cl

6 Centro de Investigación Austral Biotech, Facultad de Ciencias, Universidad Santo Tomás, Avda. Ejército 146, Santiago 8320000, Chile; ncaro@australbiotech.cl

7 Instituto de Química, Facultad de Ciencias, Pontificia Universidad Católica de Valparaíso, Av. Universidad #330, Curauma, Valparaíso2340000, Chile; mirna.melo.f@mail.pucv.cl

8 Escuela de Obstetricia y Puericultura, Facultad de medicina, Universidad de Valparaíso, Angamos 655, Reñaca, Viña del Mar 2520000, Chile; ivan.montenegro@uv.cl

* Correspondence: alejandro.madrid@upla.cl; Tel.: +56-032-250-0526 (A.M.)

NMR data for safrole and its derivatives

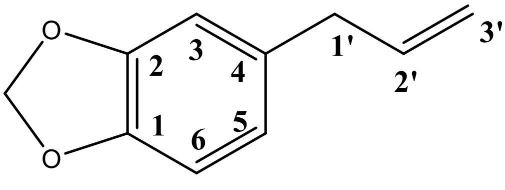

1

Figure S1. Safrole (**1**) numbered structure.

Compound 1: ^{1}H NMR: 6.73 (d, 1H, J=7.6 Hz, H-6); 6.69 (d, 1H, J=1.4 Hz, H-3); 6.64 (dd, 1H, J=1.4 and J=7.6 Hz, H-5); 5.91 (s, 2H, OCH_2O); 5.95 (ddt, 1H, J=17.0; 10.3 and 6.5 Hz, H-2'); 5.10 (m, 2H, H-3'); 3.65 (d, 2H, J=4,0 Hz, H-1'). 147.5 (C-2); 145.6 (C-1); 135.6 (C-4); 135.2 (C-2'); 121.1 (C-5); 117.0 (C-3'); 110.4 (C-3); 105.7 (OCH_2O); 102.7 (C-6); 37.6 (C-1').

Compound 4: ^{1}H NMR: 6.73 (d, 1H, J=7.6 Hz, H-6); 6.69 (d, 1H, J=1.4 Hz, H-3); 6.64 (dd, 1H, J=1.4 and J=7.6 Hz, H-5); 5.91 (s, 2H, OCH_2O); 3.65 (t, 2H, J=6.4 Hz, H-3'); 2.62 (t, 2H, J=7.4 Hz, H-1'); 1.84 (dt, 2H, J=6.4 and J=15.2 Hz, H-2'); 1.56 (b.s., 1H, OH). ^{13}C NMR: 147.5 (C-2); 145.6 (C-1); 135.6 (C-4); 121.1 (C-5); 108.8 (C-6); 108.1 (C-3); 100.7 (OCH_2O); 62.1 (C-3'); 34.4 (C-1'); 31.7 (C-2').

Compound 5: ^{1}H NMR: 7.08 (m, 2H, H-3 and H-6); 7.00 (dd, 1H, J=8.7 and J=1.5 Hz, H-5); 5.93 (ddt, 1H, J=16.8; 10.1 and 6.8 Hz, H-2'); 5.12 (dd, 1H, J=6.3 and J=1.3 Hz, H-3'α); 5.09 (t, 1H, J=1.3 Hz, H-3'β); 3.38 (d, 2H, J=6.8 Hz, H-1'); 2.28 (s, 6H, CH_3CO). ^{13}C NMR: 168.4 ($CH_3\underline{C}O$); 168.3 ($CH_3\underline{C}O$); 141.8 (C-4); 140.2 (C-2); 138.9 (C-1); 136.4 (C-2'); 126.6 (C-5); 123.3 (C-3); 123.1 (C-6); 116.6 (C-3'); 39.4 (C-1'); 20.6 (2x$\underline{C}H_3CO$).

Compound 6: ^{1}H NMR: 7.49 (s, 1H, H-6); 6.76 (s, 1H, H-3); 6.09 (s, 2H, OCH_2O); 5.95 (ddt, 1H, 1H, J=17.0, 10.3 and 6.5 Hz, H-2'); 5.10 (m, 2H, H-3'); 3.65 (d, 2H, J=4.0 Hz, H-1'). ^{13}C NMR: 151.7 (C-2); 146.5 (C-5 and C-1); 135.2 (C-2'); 132.2 (C-4); 117.0 (C-3'); 110.4 (C-3); 105.7 (OCH_2O); 102.7 (C-6); 37.6 (C-1').

Compound 7: ^{1}H NMR: 7.46 (s, 1H, H-6); 6.76 (s, 1H, H-3); 6.08 (s, 2H, OCH_2O); 3.71 (t, 2H, J=6.2 Hz, H-3'); 2.96 (dd, 2H, J=6.4 and J=8.6 Hz, H-1'); 1.90 (m, 2H, H-2') 1.50 (b.s., 1H, OH). ^{13}C NMR: 151.7 (C-2); 146.3 (C-1); 142.8 (C-5); 134.4 (C-4); 110.6 (C-3); 105.7 (C-6); 102.7 (OCH_2O); 62.0 (C-3'); 33.4 (C-2'); 30.1 (C-1').

Compound 8: ^{1}H NMR: 7.43 (s, 1H, H-6); 6.69 (s, 1H, H-3); 6.05 (s, 2H, OCH2 O); 4.07 (t, 2H, J=6.3 Hz, H-3'); 2.89 (m, 2H, H-1'); 2.03 (s, 3H, CH3); 1.93 (m, 2H, H-2'). ^{13}C NMR: 170.4 ($CH_3\underline{C}O$); 151.6 (C-2); 146.3 (C-1); 142.6 (C-5); 133.5 (C-4); 110.6 (C-3); 105.6 (OCH_2O); 102.7 (C-6); 63.4 (C-3'); 30.5 (C-2'); 29.3 (C-1'); 20.8 ($\underline{C}H_3CO$).

Compound 9: ^{1}H- NMR: 6.78 (d, 1H, J = 8.0 Hz, H-6); 6.71 (d, 1H, J = 1.9 Hz, H-3); 6.62 (dd, 1H, J = 8.0, 1.9 Hz, H-5); 5.93 (ddt, 1H, J = 16.9, 10.2 and 6.8 Hz, H-2'); 5.29 (b.s., 1H, OH); 5.21 (b.s., 1H, OH); 5.05 (m, 2H, H-3'); 3.27 (d, 2H, J = 6.7 Hz, H-1'). ^{13}C-NMR: 143.5 (C-2); 141.7 (C-1); 137.6 (C-2'); 133.2 (C-4); 121.0 (C-5); 115.7 (C-6); 115.6 (C-3); 115.3 (C-3'); 39.5 (C-1').

Compound 10: ^{1}H-NMR: 6.78 (d, 1H, J = 8.0 Hz, H-6); 6.71 (d, 1H, J = 1.9 Hz, H-3); 6.62 (dd, 1H, J = 8.0, 1.9 Hz, H-5); 5.93 (ddt, 1H, J = 16.9, 10.2 and 6.8 Hz, H-2'); 5.29 (b.s., 1H, OH); 5.21 (b.s., 1H, OH); 5.05 (m, 2H, H-3'); 3.27 (d, 2H, J = 6.7 Hz, H-1'). ^{13}C-NMR: 143.5 (C-2); 141.7 (C-1); 137.6 (C-2'); 133.2 (C-4); 121.0 (C-5); 115.7 (C-6); 115.6 (C-3); 115.3 (C-3'); 39.4 (C-1'); 20.6 (2×$\underline{C}H_3CO$).

Compound 11: ^{1}H-NMR: 7.75 (s, 1H, OH); 7.73 (s, 1H, OH); 5.69 (b.s, 1H, OH); 6.68 (m, 2H, H-3 and H-6); 6.51 (dd, 1H, J = 8.1 and J = 1.8 Hz, H-5); 3.56 (t, 2H, J = 6.5 Hz, H-3'); 2.52 (t, 2H, J = 7.7 Hz, H-

1′); 1.75 (m, 2H, H-2′). [13] C-NMR: 145.2 (C-2); 144.6 (C-1); 134.7 (C-4); 120.3 (C-5); 117.2 (C-6); 116.1 (C-3); 63.1 (C-3′); 32.1 (C-2′); 30.4 (C-1′).

Compound 12: [1] H-NMR: 7.08 (m, 2H, H-5 and H-6); 7.01 (s, 1H, H-3); 4.10 (t, 2H, J = 6.5 Hz, H-3′); 2.68 (t, 2H, J = 7.8 Hz, H-1′); 2.26 (s, 6H, CH_3CO); 2.05 (s, 3H, CH_3CO); 1.98 (m, 2H, H-2′). [13] C-NMR: 171.1 (CH_3CO); 168.4 (CH_3CO); 168.3 (CH_3CO); 141.9 (C-2); 140.2 (C-1); 140.1 (C-4); 126.5 (C-5); 123.2 (C-3); 123.1 (C-6); 63.6 (C-3′); 31.6 (C-2′); 29.9 (C-1′); 20.9 (OCH_3); 20.6 (2 × $O\underline{C}H_3$).
